# Editorial: Green technologies and bioactive profiling in aromatic and medicinal plants

**DOI:** 10.3389/fphar.2026.2001873

**Published:** 2026-10-07

**Authors:** Hanen Najjaa, Patricia Rijo

**Affiliations:** 1 Laboratory of Pastoral Ecosystems and Valorization of Spontaneous Plants and Microorganisms, Institute of Arid Regions, Medenine, Tunisia; 2 Laboratory of Natural Bioactives (Bio.Natural), CBIOS Lusófona’s Research Center for Biosciences and Health Technologies, Lisbon, Portugal; 3 Research Institute for Medicines (iMed.ULisboa), Faculty of Pharmacy, Universidade de Lisboa, Lisbon, Portugal; 4 Centro de Química Estrutural, Institute of Molecular Sciences, Universidade de Lisboa, Lisboa, Portugal; 5 Faculty of Biology and Environmental Protection University of Lodz, Lodz, Poland

**Keywords:** aromatic and medicinal plants, bioactive compound, green technologies, pharmacology, phytochemistry

Aromatic and medicinal plants are a rich source of structurally diverse natural compounds with applications in human health, food, agriculture, and biotechnology. Their growing scientific and economic relevance is closely linked to the need for sustainable strategies that preserve plant resources while improving the recovery, characterization, and utilization of their bioactive constituents. In this context, green technologies, advanced phytochemical profiling, and integrated biological evaluation offer complementary approaches that help bridge plant biodiversity with scientifically supported and sustainable applications.

The Research Topic *“Green Technologies and Bioactive Profiling in Aromatic and Medicinal Plants”* brings together eight contributions that collectively explore these interconnected dimensions. Rather than considering extraction, chemical characterization, processing, and biological activity as separate steps, the contributions illustrate the importance of integrating these approaches to improve the valorization of aromatic, medicinal, and spontaneous plants, including resources originating from arid and underexplored environments.

A key perspective emerging from this Research topic is the relevance of sustainable processing and resource-efficient technologies. The preservation of *Terfezia boudieri* and *Tirmania nivea* demonstrates that processing choices can substantially influence nutritional, phytochemical, and antioxidant characteristics, highlighting preservation as a critical component of the valorization chain (Ghanney et al.). Similarly, ultrasound-assisted extraction of *Peucedanum ostruthium* leaves demonstrates the potential of innovative extraction combined with response surface methodology to transform an underutilized plant fraction into a source of biologically active compounds (Chen et al.). Together, these studies emphasize that sustainable valorization should address not only what compounds are present, but also how plant resources can be processed efficiently and responsibly.

A second major dimension is the integration of phytochemical profiling with biological and computational approaches. The studies on *Anisosciadium lanatum*, *Farsetia aegyptia*, and *Zilla spinosa* illustrate this multidisciplinary strategy by combining chromatographic characterization with antioxidant, antimicrobial, enzymatic, cytotoxic, antibiofilm, molecular docking, and, where applicable, ADME analyses (Besbes et al.; Besbes et al.). Such integration can strengthen the interpretation of plant bioactivity by connecting chemical composition with functional responses and potential molecular interactions.

The Research Topic further demonstrates how plant-derived bioactive resources may contribute to contemporary challenges in agriculture and infectious disease control. The investigation of four spontaneous plants from Tunisian arid regions provides evidence of concentration-dependent allelopathic effects against selected weeds and crops, supporting further exploration of plant metabolites as potential resources for more sustainable weed management (Harabi et al.). In parallel, research on Tunisian *Thymus vulgaris* essential oil and carvacrol against carbapenem-resistant *Acinetobacter baumannii* highlights the potential of plant-derived compounds as complementary candidates in strategies addressing antimicrobial resistance, particularly through observed synergistic interactions with imipenem (Jilani et al.).

The health-related contributions extend this perspective from biological screening toward therapeutic and mechanistic investigation. The study of *Curcuma caesia* provides preclinical evidence for effects on male reproductive parameters while demonstrating the importance of dose-dependent responses in evaluating medicinal plant preparations (Suriani et al.). Complementing this work, the study of six-methoxyflavone investigates molecular mechanisms associated with melanoma progression, including circPIAS1 regulation, PTBP1, PI3K-AKT signaling, ferroptosis-related pathways, and interaction with immunotherapeutic treatment (Guo et al.).

Collectively, these contributions illustrate a broader transition in aromatic and medicinal plant research: from the identification of traditionally used or underexplored resources toward integrated, technology-supported and evidence-based valorization. Green extraction and preservation technologies can improve resource efficiency; advanced phytochemical profiling can reveal chemical diversity; biological assays can establish functional potential; and computational approaches can provide complementary insights into possible mechanisms. Importantly, the diversity of applications represented in this Research Topic, from functional nutrition and healthcare to sustainable agriculture and antimicrobial research, demonstrates the cross-sector relevance of plant bioresources.

Future research should increasingly connect these approaches with extract standardization, reproducibility, safety and toxicological assessment, sustainable cultivation and harvesting, and validation in relevant biological models. Such integration will be essential for translating promising laboratory findings into reliable and sustainable products and technologies while ensuring the responsible use of plant biodiversity.

Through this Research Topic, we aim to encourage interdisciplinary research and international collaboration at the interface of plant science, phytochemistry, green technologies, pharmacology, food science, microbiology, and sustainable agriculture. We hope that these contributions will advance the scientific valorization of aromatic and medicinal plants and stimulate further research toward sustainable solutions based on plant-derived bioactive resources.

